# Laryngeal Synovial Sarcoma: A Rare Clinical Entity

**DOI:** 10.1155/2013/578606

**Published:** 2013-07-08

**Authors:** Clair Saxby, Ron Bova, Melanie Edwards

**Affiliations:** ^1^St Vincents Hospital, Sydney, NSW 2010, Australia; ^2^St Vincents Clinic, Sydney, NSW 2010, Australia

## Abstract

*Introduction*. Synovial sarcomas (SS) are aggressive malignant soft tissue tumours that are thought to arise from pluripotent mesenchymal cells. *Clinical Report*. A 20-year-old male presented with an acute onset of respiratory stridor. Computer tomography scanning confirmed a mass arising from the left supraglottic larynx and an emergency tracheostomy was performed. A diagnosis of biphasic synovial sarcoma was formed. A total laryngectomy and left hemithyroidectomy was performed in conjunction with a left modified radical neck dissection. The patient received adjuvant chemotherapy followed by a course of radiotherapy and remains alive and disease free at 18 months after treatment. *Discussion*. Prognosis for patients with SS is related to primary tumour extent, grade, and size. The presence of the diagnostic translocation, t(X;18), is being targeted and hopefully will lead to the development of new therapeutics (Guadagnolo et al., 2007). *Conclusion*. Laryngeal SS remains a rare and poorly understood entity. A multidisciplinary approach to treatment is essential and long-term followup is imperative.

## 1. Introduction 

Sarcomas represent 1% of all head and neck malignancies [1]. Only 10% of soft tissue sarcomas are synovial in type. Synovial sarcomas (SS) are aggressive malignant soft tissue tumours that are thought to arise from pluripotent mesenchymal cells and usually involve large joints within the lower extremities [1]. Only 3% of cases arise in the head and neck [2]. The most common site is the hypopharynx with the larynx being the least common site [3]. The name SS arises from the histological appearance which resembles a synovial membrane [4]. There have been very few cases of laryngeal SS reported in the literature.

## 2. Case Report

**Figure 1:**
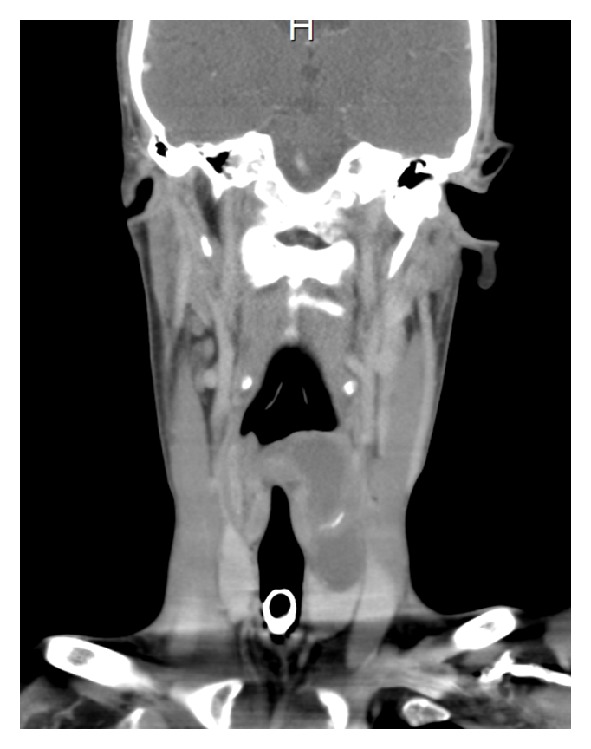
CT scan head and neck, coronal view, well circumscribed mass arising from the left supraglottic larynx extending into the left thyroid lobe.

**Figure 2:**
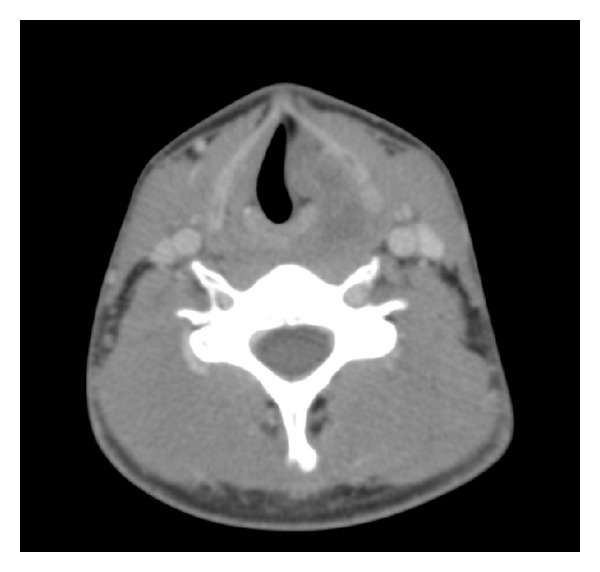
CT scan of neck, axial view, showing laryngotracheal deviation.

An otherwise fit and well nonsmoking 20-year-old male presented with an acute onset of respiratory stridor associated with a sore throat and odynophagia. Flexible nasendoscopy revealed a large well circumscribed mass in the left aryepiglottic fold which was causing some degree of laryngeal inlet obstruction. Computer tomography (CT) scanning confirmed a large cystic mass measuring 7.5 cm × 3.6 cm arising from the left supraglottic larynx extending through the cricothyroid membrane into the left thyroid lobe (Figure 1). There was associated laryngotracheal deviation (Figure 2). An emergency tracheostomy was performed and an open biopsy of the left thyroid mass was obtained. Histopathological examination revealed a high grade biphasic tumour composed of nests and ribbons of epithelioid and plump spindle cells with areas of necrosis. Fluorescent in situ hybridisation (FISH) confirmed the diagnostic t(X;18) translocation for synovial sarcoma. A diagnosis of biphasic SS, grade three, was made.

A positron emission tomography with fluoro-deoxyglucose (FDG-PET) scan revealed avid isotope uptake in the left larynx and thyroid, with some mild uptake in several upper left cervical lymph nodes. There was no evidence of distant metastatic disease.

**Figure 3:**
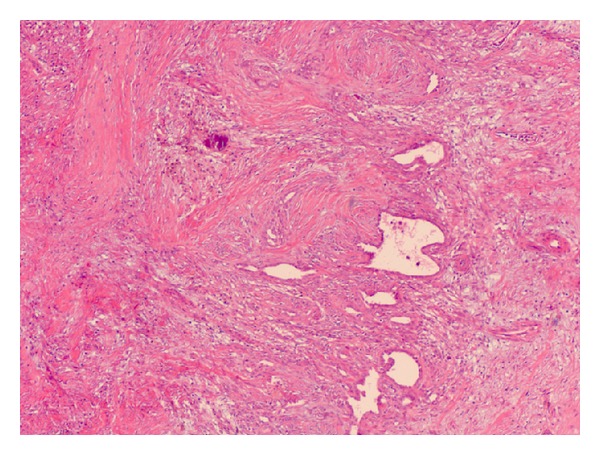
Low power view of tumour with glandular spaces lined by epithelial cells in a background of plump spindle cells with stromal calcification (Haematoxylin and eosin, ×4).

**Figure 4:**
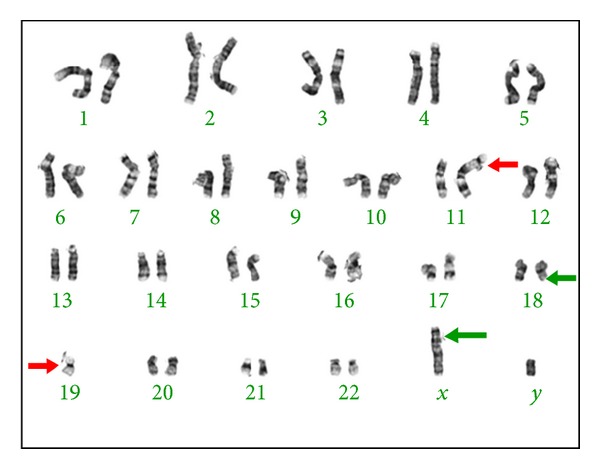
Karyotype showing characteristic X;18 translocation (green arrows) as well as association of chromosome 19 with telomere of chromosome 11 (red arrows).

A total laryngectomy and left hemithyroidectomy were performed in conjunction with a left modified radical neck dissection. The final histopathology confirmed SS showing characteristic biphasic histology (Figure 3) with clear margins, and none of the 25 cervical lymph nodes were involved. Conventional cytogenetics performed on fresh tissue once again showed the diagnostic t(X;18) translocation as well as other cytogenetic abnormalities (Figure 4). The patient then received adjuvant chemotherapy (Adriamycin and Ifosfamide for six cycles) followed by a full course of radiotherapy. The patient remains alive and disease free at 18 months after treatment.

## 3. Discussion

The American Cancer Society estimates that in 2012 there will be about 12,360 new cases of laryngeal cancer diagnosed [5]. Squamous cell carcinoma accounts for over 90% of all laryngeal cancers [1]. Laryngeal SS is an extremely rare form of laryngeal carcinoma. The median age of patients at diagnosis of SS is the third decade of life and there is a mild male dominance [6]. 

SS acquired its name due to its microscopic resemblance to developing synovium but is immunophenotypically and ultrastructurally distinct from normal synovium, only rarely arising in joint cavities, and usually occurs in association with para-articular regions of the extremities, with no relation to synovial structures [7]. Except in the paediatric population, sarcomas occur uncommonly in the head and neck region [1] and head and neck SS is extremely rare with less than 20 cases in the literature arising from the larynx [8]. 

Histologically, SS can be divided into two main groups: biphasic and monophasic. Both variants contain a population of monomorphic spindle cells arranged in fascicles with tapering nuclei and pale, ill-defined cytoplasm set in a variably collagenous stroma. In addition, classic biphasic lesions contain glandular structures lined by well-differentiated cuboidal to columnar epithelium. A branching hemangiopericytoma-like vascular pattern is characteristic and a common finding in both types is the presence of stromal calcification, which ranges from focal to extensive and is an important diagnostic clue [9]. 

Biphasic lesions generally pose no diagnostic difficulty but those in unusual locations may raise a differential diagnosis of other biphasic tumours such as carcinosarcoma, malignant mesothelioma, and malignant peripheral nerve sheath tumour (MPNST), which rarely has glandular elements, particularly those arising in patients with neurofibromatosis type 1 [7]. 

Immunohistochemically, SS is characterised by coexpression of mesenchymal and epithelial markers (cytokeratins and epithelial membrane antigen) [3]. About 30% of SS stain with S-100, which can cause confusion with MPNST but EMA staining is infrequent in MPNST [9]. There is some histological overlap with malignant mesothelioma as more than 50% of SS stain with calretinin; however, unlike mesothelioma, they are usually Ber-Ep4 positive and WT1 negative [7]. 

Cytogenetics contribute greatly to the diagnosis of SS as 90% harbour a specific translocation between the SYT gene on chromosome 18 and either the SSX1 or SSX2 gene on the X chromosome [1]. The type of fusion product correlates with the histological pattern; those with SYT-SSX1 are usually biphasic and those with SYT-SSX2 are monophasic. Genetic testing is particularly useful in the poorly differentiated tumours, which may be difficult to distinguish from other spindle cell and round cell sarcomas by other means [7].

Prognosis for patients with SS is related to primary tumour extent, tumour grade, and size [6]. The 5-year survival rate has been reported to be approximately 70% to 80%, and the 10-year survival rate approximately 50% [10]. The optimal treatment of SS is multimodal. Radical surgical excision is generally accepted as the mainstay of therapy [1]. Adjuvant chemotherapy has been utilised for high grade synovial sarcoma. Doxorubicin and ifosfamide have been shown to demonstrate improvement in disease specific survival in the treatment of soft tissue sarcomas [11, 12]. Adjuvant radiotherapy has also been shown to reduce local recurrence rates but not overall survival rates [6, 13]. 

Disease recurrence is a significant problem, with up to 45% of patients with head and neck SS developing a local recurrence and 33% developing distant metastatic disease [14]. The presence of the diagnostic translocation, t(X;18), is being targeted and hopefully will lead to further understanding of the tumors' biology and the development of new therapeutics [15].

## 4. Conclusion

Our case adds to current literature of laryngeal SS which remains a rare and relatively poorly understood entity. There are no well-established risk factors to enable a screening plan to be recommended or management protocols to be constructed. A multidisciplinary approach to diagnosis and treatment is essential to improve the management of SS. Long-term followup is imperative due to the relatively high locoregional and metastatic recurrence rates [16]. Future research will hopefully improve our understanding of the aetiology and genetic basis of this unusual malignancy which will hopefully translate to refinement of treatment protocols and improved survival in patients diagnosed with SS. 

## 5. Summary

- Synovial sarcomas are aggressive malignant soft tissue tumours that usually involve large joints within the lower extremities. They are extremely rare within the head and neck.
- Our case report describes a young male with a biphasic laryngeal synovial sarcoma who remains disease free 18 months after treatment.
- Cytogenetics contribute greatly to the diagnosis of synovial sarcoma as 90% have the (X;18) trans-location. This is being targeted and will hopefully lead to the development of new therapeutics.
- A multidisciplinary approach to management and long-term followup is essential due to the high locoregional and metastatic recurrence rates.
